# Seltene Erkrankungen in den Daten sichtbar machen – Kodierung

**DOI:** 10.1007/s00103-022-03598-9

**Published:** 2022-10-14

**Authors:** Tamara Martin, Kathrin Rommel, Carina Thomas, Jutta Eymann, Tanita Kretschmer, Reinhard Berner, Min Ae Lee-Kirsch, Helge Hebestreit

**Affiliations:** 1 Zentrum für Seltene Erkrankungen und Institut für Medizinische Genetik und Angewandte Genomik, Universität und Universitätsklinikum Tübingen, Tübingen, Deutschland; 2 Abteilung K – Kodiersysteme, Fachgebiet – K4 Orphanet Deutschland, Bundesinstitut für Arzneimittel und Medizinprodukte (BfArM), Bonn, Deutschland; 3 Zentrum für Seltene Erkrankungen, Universität und Universitätsklinikum Tübingen, Tübingen, Deutschland; 4 Klinik und Poliklinik für Kinder- und Jugendmedizin und UniversitätsCentrum für Seltene Erkrankungen (USE), Universitätsklinikum Carl Gustav Carus der TU Dresden, Dresden, Deutschland; 5 Zentrum für Seltene Erkrankungen – Referenzzentrum Nordbayern, Universitätsklinikum Würzburg, Josef-Schneider-Str. 2, 97080 Würzburg, Deutschland

**Keywords:** Seltene Erkrankung, ORPHAcode, Alpha-ID-SE, Human Phenotype Ontology, Diagnose, Rare diseases, ORPHAcode, Alpha-ID-SE, Human phenotype ontology, Diagnosis

## Abstract

Seltene Erkrankungen (SE) werden durch die im deutschen Gesundheitssystem verwendete Diagnosenklassifikation ICD-10-GM (International Statistical Classification of Diseases and Related Health problems, 10th Revision, German Modification) nur zu einem kleinen Teil eindeutig erfasst. Daher sind Aussagen zur Häufigkeit von SE sowie zum speziellen Versorgungs- und Finanzierungsbedarf nicht möglich, was zu einer lückenhaften Datenlage als Entscheidungsgrundlage für Krankenkassen, Leistungserbringer und Gesundheitspolitik führt. Das Fehlen exakter Informationen behindert auch die wissenschaftliche Arbeit. Daher wird deutschlandweit ab 2023 die Verwendung der Alpha-ID-SE-Datei und der ORPHAcodes für die spezifische Erfassung von SE bei stationären Fällen verpflichtend.

Die Alpha-ID-SE-Datei verknüpft die ICD-10-GM-Kodes mit den international anerkannten ORPHAcodes für die Diagnose von SE. Kommerzielle Anbieter stellen zunehmend die benötigten IT-Tools zur Kodierung von SE zur Verfügung. An mehreren Universitätskliniken mit Zentren für SE wurden Lösungen etabliert, die eine vollständige Kodierung gewährleisten sollen. Hierzu gehören finanzielle Anreize für die kodierenden Bereiche, konkrete Nachfragen nach dem Vorliegen einer SE beim Kodiervorgang und eine semiautomatische Kodierung bei Patient*innen, die schon einmal mit einer SE an der Einrichtung betreut worden waren. Eine Kombination der verschiedenen Ansätze verspricht die höchste Wahrscheinlichkeit einer vollständigen Kodierung.

Für ein umfängliches Bild der SE im Gesundheitssystem und um dem speziellen Versorgungs- und Finanzierungsbedarf besser Rechnung tragen zu können, wäre auch im ambulanten Bereich eine möglichst spezifische und eindeutige Kodierung wünschenswert. Für komplexe SE und bisher undiagnostizierte Patient*innen wird zusätzlich eine strukturierte Erfassung des Phänotyps benötigt.

## Abstract (en)

The ICD-10-GM coding system used in the German healthcare system only captures a minority of rare disease diagnoses. Therefore, information on the incidence and prevalence of rare diseases as well as necessary (financial) resources for the expert care required for evidence-based decisions by health insurers, care providers, and politicians are lacking. Furthermore, the missing information complicates and sometimes even precludes the generation of scientific knowledge on rare diseases. Therefore, starting in 2023, all in-patient cases in Germany with a rare disease diagnosis must be coded by an ORPHAcode using the Alpha-ID-SE file.

The file Alpha-ID-SE links the ICD-10-GM codes to the internationally established ORPHAcodes for rare diseases. Commercially available software tools progressively support the coding of rare diseases. In several centers for rare diseases linked to university hospitals, IT tools and procedures were established to realize a complete coding of rare diseases. These include financial incentives for the institutions providing rare disease codes, systematic queries asking for rare disease codes during the coding process, and a semi-automated coding process for all patients with a rare disease previously seen at the institution. A combination of the different approaches probably results in the most complete coding.

To get the complete picture of rare disease epidemiology and care requirements, a specific and unique coding of out-patient cases is also desirable. Furthermore, a structured reporting of phenotype is required, especially for complex rare diseases and for yet undiagnosed cases.

## Einleitung

Im deutschen Gesundheitssystem erfolgt die Kodierung von Erkrankungen bzw. Diagnosen mit der Internationalen statistischen Klassifikation der Krankheiten und verwandter Gesundheitsprobleme ICD-10-GM (GM = German Modification). Allerdings erlaubt die ICD-10-GM-Kodierung nur für wenige Seltene Erkrankungen (SE) eine eindeutige Identifikation. Denn für die meisten SE ist im ICD-10-GM nur ein Kode vorgesehen, dem gleichzeitig weitere und in der Regel häufige Erkrankungen zugeordnet sind [1]. Hierdurch sind Menschen mit SE in den aktuell erfassten Gesundheitsdaten oft nicht als solche zu erkennen. Mit anderen Worten: Die Informationen, die von den Leistungserbringern bzgl. der Diagnosen ihrer Patient*innen gesammelt und z. B. an die Krankenkassen übermittelt werden, sind bzgl. der Abbildung von SE unzulänglich. Dadurch lassen sich für die Hauptakteure im Gesundheitswesen – also Krankenkassen, Leistungserbringer und Gesundheitspolitik – weder Aussagen zur Häufigkeit solcher Erkrankungen noch zum speziellen Versorgungs- und Finanzierungsbedarf machen. Aber auch für Betroffene selbst kann im deutschen Gesundheitssystem nicht evidenzbasiert dargestellt werden, welche Institution häufig einzelne SE oder bestimmte Gruppen von SE versorgt und damit eine besondere Expertise besitzt. Und nicht zuletzt sind exakte Diagnose-Informationen auch für Forschungsfragen, von Versorgungsforschung bis hin zur Planung klinischer Studien, hochrelevant.

Eine durch die internationale Referenzdatenbank für SE „Orphanet“ entwickelte Nomenklatur erlaubt die eindeutige Kodierung von SE mittels sogenannter ORPHAcodes bzw. Orpha-Kennnummern [2]. Mit Inkrafttreten des Digitale-Versorgung-und-Pflege-Modernisierungs-Gesetz (DVPMG) wurde die Grundlage geschaffen, um die eindeutige Kodierung von SE mithilfe der ORPHAcodes zusätzlich zur ICD-10-GM-Kodierung im stationären Bereich verbindlich vorzugeben. Hier werden die Kodierung und Übermittlung der ORPHAcodes an die Krankenkassen ab 2023 verpflichtend, sofern ICD-10-GM-Kode und ORPHAcode für die jeweilige SE enthalten sind. Die Zuordnung zwischen ICD-10-GM-Kode und ORPHAcode für die einzelne SE erfolgt dabei über die Datei Alpha-ID-SE. Der vorliegende Beitrag adressiert die Hintergründe und Implementierung der Kodierung mittels ORPHAcodes mit einem besonderen Blick auf die Situation in Deutschland und stellt anhand von Umsetzungsbeispielen aus der Praxis erste Erfahrungen mit einer solchen Kodierung vor.

Um die interindividuellen Unterschiede in Manifestation oder Verlauf von SE zu erfassen oder bei Menschen mit letztendlich unklarer Diagnose die Symptomatik zu beschreiben, ist eine symptombezogene Kodierung zusätzlich zur Angabe eines ORPHAcodes erforderlich. Für SE wird die Human Phenotype Ontology (HPO; [3]) für diesen Zweck in vielen Projekten eingesetzt und findet an einigen deutschen Standorten bereits Einzug in die Routinedaten. Die HPO bietet ein standardisiertes Vokabular für phänotypische Veränderungen, die im Rahmen von Erkrankungen beim Menschen beobachtet werden, und ordnet diesen eine HPO-ID-Ziffer zu (z. B. HP:0010704 für eine Verwachsung bzw. Syndaktylie der Finger 1 und 2). Eine genauere Erläuterung findet sich weiter unten im Abschnitt „Erfassung des Phänotyps“. Auch diese erweiterte Kodierung von SE wird in diesem Beitrag angesprochen.

## Seltene Erkrankungen und ORPHAcodes

**Figure 1:**
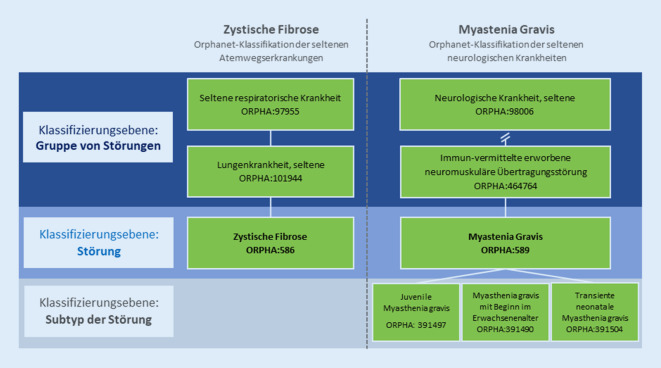

Die Kodierung von SE kann nur dann erfolgreich sein, wenn diese Krankheiten im Vorfeld durch eine systematische Dokumentation beschrieben und durch eine wissenschaftliche Zuordnung klassifizierbar sind. Der Grundstein hierzu wurde durch das europäische Projekt Orphanet gelegt, das seit 1997 als internationale Referenzdatenbank über SE und Arzneimittel für SE (Orphan Drugs) informiert [4]. Die von der Europäischen Kommission geförderte Ressource stellte erstmalig eine umfassende Inventarliste von SE zur Verfügung, die auf Grundlage systematischer Literaturrecherchen beständig erweitert wird. Das Verzeichnis der SE ist eingebunden in die eigens konzipierte Orphanet-Nomenklatur, die unter Verwendung des sogenannten ORPHAcodes (Orpha-Kennnummer) eine spezifische Kodierung von SE ermöglicht (Abb. 1).

Die Einträge der Orphanet-Datenbank sind mit weiteren Terminologien und Kodiersystemen gemappt, so dass eine Interoperabilität zwischen verschiedenen Datensystemen ermöglicht wird. Das Mapping umfasst systematische Zuordnungen u. a. zu OMIM^1^, ICD-10-WHO^2^, UMLS^3^, SNOMED CT^4^, aber auch Thesauri, wie z. B. MeSH^5^ und MedDRA^6^. Alle Dateien der Orphanet-Terminologie inklusive Verweisdaten sind öffentlich und zum Download frei verfügbar auf der Online-Plattform Orphadata.org [5]. Die aktuell verfügbare Datei (ORPHA NOMENCLATURE PACK, Juli 2021) enthält 6165 aktive ORPHAcodes, die eine Kodierung von SE auf der von Orphanet definierten Ebene der Störung ermöglichen.

Im Jahr 2009 wurde die Kodierung von SE in der Empfehlung des Rates der europäischen Kommission für eine Maßnahme im Bereich von SE [6] angeraten, um sicherzustellen, dass „… seltene Krankheiten in geeigneter Weise kodiert werden und in allen Gesundheitsinformationssystemen auffindbar sind …“. Da weniger als 500 der SE in der ICD-10 mit einem präzisen Diagnoseschlüssel belegt sind, erwies sich die Nutzung der ICD-10, die weltweit zur Verschlüsselung von Diagnosen dient, für diesen Zweck als unzureichend [7]. Bei der Beurteilung dieser Zahl muss auch bedacht werden, dass einzelnen SE wie der Mukoviszidose sogar mehrere ICD-10-GM-Kodes zugeordnet sind. Die tatsächliche Zahl individueller SE, die im ICD-10-GM abgebildet sind, liegt deutlich niedriger.

Im Rahmen des Revisionsprozesses der ICD wurde 2009 eine beratende Gruppe für SE, die „WHO Topic Advisory Group for Rare Diseases“ etabliert, die eine Implementierung der SE in die zukünftige ICD-11-Fassung unterstützen sollte. Im Jahr 2015 wurde eine erste Beta-Version des ICD-11 veröffentlicht, die 5400 SE von damals 6954 in Orphanet gelisteten Krankheiten enthielt [8]. Aufgrund der sehr komplexen und umfangreichen Aktualisierungen konnte der generelle Revisionsprozess jedoch nicht im gesetzten Zeitrahmen umgesetzt werden und nur wenige Mitgliedsländer zeigten eine potenzielle Bereitschaft, die Beta-Version im Feldtest umzusetzen. Letztlich wurde die ICD-11 erst im Mai 2019 verabschiedet und trat im Januar 2022 in Kraft. Ein konkreter Zeitpunkt zur Einführung der ICD-11 zum Zwecke der Morbiditätskodierung in Deutschland steht bisher jedoch nicht fest und wird aufgrund der hohen Integration der ICD-10-GM im deutschen Gesundheitswesen und der damit verbundenen Komplexität voraussichtlich noch Jahre in Anspruch nehmen. Als Reaktion auf die damals verzögerten Entwicklungen wurde im November 2014 von der „European Commission Expert Group on Rare Diseases“ eine Maßnahme verabschiedet, die eine Verwendung von ORPHAcodes zusätzlich zu den ICD-10-Codes für SE empfahl. Die Umsetzung dieser Empfehlung wurde unter deutscher Beteiligung durch das europäische Projekt RD-Action (2014–2020) und RD-Code (2019–2021) begleitet und aktuell von dem Projekt OD4RD (2022–2023) unter Mitwirkung des Bundesinstituts für Arzneimittel und Medizinprodukte (BfArM) fortgeführt [9–11].

### Situation in Deutschland

Auch in Deutschland wurde frühzeitig nach Möglichkeiten gesucht, um die SE bereits vor Einführung der ICD-11 in nationalen Forschungssystemen und Registern abzubilden. Im Rahmen des Nationalen Aktionsplans für SE [12] initiierte das Nationale Aktionsbündnis für Menschen mit Seltenen Erkrankungen (NAMSE) im Jahr 2013 das vom Bundesministerium für Gesundheit geförderte Projekt „Kodierung von Seltenen Erkrankungen“ (2013–2019; [9]). Dieses Vorhaben sollte durch eine verknüpfte Kodierung von ICD-10-GM und ORPHAcode mithilfe der Datei Alpha-ID des Deutschen Instituts für Medizinische Dokumentation und Information (DIMDI) eine ressourcenschonende Lösung eröffnen, um SE in digitalen Datensystemen sichtbar zu machen. Dabei enthält jeder Eintrag des alphabetischen Verzeichnisses eine fortlaufende, stabile, nichtsprechende Identifikationsnummer und repräsentiert somit einen nichtklassifizierenden Diagnosencode.

In der Version 2015 veröffentlichte das DIMDI erstmals eine erweiterte Variante der Alpha-ID, die sog. Alpha-ID-SE, die neben dem ICD-10-GM-Kode zusätzlich ORPHAcodes für Diagnosebezeichnungen von SE enthält. Die Alpha-ID-SE wird inzwischen jährlich vom BfArM veröffentlicht und aktualisiert.^7^ Die aktuelle Datei Version 2022 enthält mehr als 85.000 Diagnosebezeichnungen von Krankheiten sowie 5796 spezifische ORPHAcodes, die eine parallele Kodierung von SE ermöglichen. Durch die Verknüpfung von Alpha-ID, Erkrankungsbegriff, ICD-10-GM-Kode und ORPHAcode erhalten Kodierende in einem Schritt alle notwendigen Informationen zur Kodierung. Mit Inkrafttreten des DVPMG am 09.06.2021 wurde die gesetzliche Grundlage geschaffen, um die eindeutige Kodierung von SE mithilfe der Orpha-Kennnummer zusätzlich zur Schlüsselnummer der ICD-10-GM im stationären Bereich umzusetzen [13]. Die Kodierung anhand der Datei Alpha-ID-SE wird ab dem Jahr 2023 verpflichtend, sofern ICD-10-GM-Kode und ORPHAcode für die jeweilige SE enthalten sind.

### Orphanet-Nomenklatur

Die Orphanet-Nomenklatur wird in Englisch erstellt und aktuell in 8 weitere Sprachen (CZ, NL, FR, DE, IT, PL, PT, ES) übersetzt. Jeder Entität in der Orphanet-Nomenklatur ist ein nach Zufallsprinzip generierter ORPHAcode zugeordnet, der als eindeutiger, zeitlich stabiler und nicht wiederverwendbarer numerischer Identifikator dient. Jeder Entität ist ein Vorzugsbegriff zugeordnet. Weitere Bestandteile der Nomenklatur sind Synonyme (deren Anzahl nicht festgelegt ist bzw. je nach Übersetzungssprache variiert) und ggf. Akronyme sowie eine textliche Kurzdefinition der jeweiligen Entität. Die Orphanet-Nomenklatur ist nach medizinischen Fachgebieten gegliedert, um dem multidimensionalen Charakter von SE Rechnung zu tragen. Jede Entität kann in Abhängigkeit ihrer klinischen Ausprägung mehreren Fachgebieten angehören und somit in mehrere Klassifikationen aufgenommen werden.

Die Orphanet-Nosologie ist vordergründig in solche Systeme unterteilt, die der Organisation der übergeordneten medizinischen Fachgebiete entsprechen und die häufig die Beteiligung der Organsysteme widerspiegeln. Darüber hinaus können praxisabhängig jedoch auch hereditäre, anatomisch-klinische, bildgebende, histologische oder sogar mechanistische Kriterien in der Orphanet-Klassifikation Anwendung finden. Diese Unterteilungen führen zu einer Verzweigung entsprechend der Typologie der klinischen Entitäten, von der übergeordneten Störung bis hin zur kleinsten Einheit, den Subtypen (Abb. 1).

Eine Gruppe von Störungen repräsentiert hierbei eine zusammenfassende klinische Einheit, die durch eine Reihe gemeinsamer Merkmale mehrerer Störungen definiert ist. Es kann sich um eine bestimmte Kategorie oder eine klinische Gruppe handeln. Eine Störung meint eine klinische Entität, die durch eine Reihe von phänotypischen Anomalien mit einer homogenen Entwicklung definiert ist und eine definitive klinische Diagnose ermöglicht. Es kann sich um eine Krankheit, eine Fehlbildung oder ein klinisches Syndrom, eine morphologische oder biologische Anomalie oder eine besondere klinische Situation bei einer Krankheit oder einem Syndrom handeln. Auf der untersten Ebene befindet sich der Subtyp, der eine weitere Unterteilung einer Störung darstellt (z. B. klinischer, ätiologischer oder auch histopathologischer Subtyp). Für die Kodierung der Patient*innen und für statistische Erhebungszwecke empfiehlt Orphanet die Zuordnung der Krankheiten auf Ebene der Störung.

### Kommerzielle Systeme zur Unterstützung der Kodierung mit ORPHAcodes

Das Schaffen der technischen Voraussetzungen für die Umsetzung der ab 2023 gesetzlich verpflichtenden Kodierung von SE im stationären Bereich liegt in der Hand der kommerziellen Anbieter von Krankenhausinformationssystemen und Kodiersoftware-Tools. Ende 2021 erweiterte der Anbieter SAP im seinem Krankenhausinformationssystem IS‑H die Diagnosentabelle NDIA um Felder für Alpha-IDs und ORPHAcodes. Diese Felder können durch direkte Eingabe der entsprechenden Kodes oder über die Schnittstelle aus einer integrierten Kodiersoftware heraus befüllt werden, z. B. durch Auswahl einer Diagnose im Reiter „Seltene“ in der Kodiersoftware ID DIACOS® von ID Berlin. Der Anbieter SAP wird zur Umsetzung der gesetzlichen Vorgaben noch weitere Voraussetzungen schaffen. Auch andere kommerzielle Anbieter, wie z. B. Dedalus ORBIS und die Kodiersoftware 3M KODIP, bieten die Möglichkeit, SE mit Alpha-IDs und ORPHAcodes zu kodieren. Daneben gibt es auch einige hausinterne Lösungen einzelner Unikliniken zur Abbildung von SE.

## Erfassung des Phänotyps

Bei Menschen mit einer SE ist eine strukturierte, systematische Beschreibung des Phänotyps unerlässlich für Diagnosestellung und Behandlung, insbesondere bei noch nicht diagnostizierter SE oder einem komplexen Phänotyp. Eine Kodierung des Phänotyps ist für die Diagnosefindung v. a. mittels digitaler Werkzeuge relevant, aber auch notwendig für eine Suche nach ähnlichen Fällen zur gegenseitigen konsiliarischen Beratung z. B. bezüglich einer möglichen Behandlung.

Die Human Phenotype Ontology (HPO) erlaubt sowohl die Kodierung von Fehlbildungen wie einem Vorhofseptumdefekt als auch die von Symptomen wie Schmerzen oder Erschöpfung. Für Menschen mit einer (noch) nicht diagnostizierten SE gehört die Beschreibung des Phänotyps mittels HPO sowie des Genotyps anhand der Nomenklatur der Human Genome Variation Society zum „Set of common data elements“ der Europäischen Plattform für die Registrierung Seltener Erkrankungen [14]. Eine Beschreibung des Phänotyps mittels HPO spielt weiterhin für die Auswertung genomischer Daten eine wichtige Rolle, zum Beispiel für die aus dem Projekt TRANSLATE-NAMSE entwickelten Selektivverträge nach § 140a Fünftes Buch Sozialgesetzbuch (SGB V) zur Durchführung von Fallkonferenzen und Exomsequenzierung bei Patient*innen mit unklarer Diagnose und dem Verdacht auf eine Seltene Erkrankung. Die Beschreibung des Phänotyps mittels HPO wird wohl auch für das Modellvorhaben § 64e SGB V zur umfassenden Diagnostik und Therapiefindung mittels Genomsequenzierung bei seltenen und bei onkologischen Erkrankungen hoch relevant werden.

In Zukunft können auch andere Systeme wie SNOMED CT zunehmend wichtig für die Kodierung des Phänotyps bei SE werden. Der aktuelle SNOMED-Mapping-File „SNOMED CT-ORPHANET MAP RELEASE“ enthält 5652 ORPHAcodes, die mit den entsprechenden SNOMED-Konzepten gemappt sind [5].

## Umsetzung der Kodierung von SE in der Praxis

Den oben dargestellten gesetzlichen Entwicklungen, der Forderung nach der Dokumentation von ORPHAcodes durch die Fach-(Typ-B-)Zentren für Seltene Erkrankungen im Anforderungskatalog des NAMSE sowie den Aktivitäten der Collaboration on Rare Diseases der Medizininformatik-Initiative (CORD-MI) ist es zu verdanken, dass in den vergangenen Jahren zumindest einige wenige Unikliniken die Kodierung von ORPHAcodes etabliert haben. Im Folgenden werden drei Beispiele vorgestellt, welche frühzeitig interne Lösungen zur Kodierung implementierten.

### Universitätsklinikum Dresden

**Figure 2:**
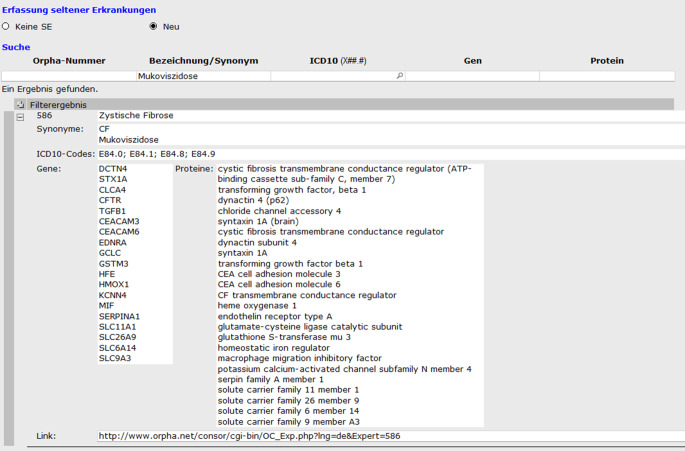

Um eine vollständige und prospektive Kodierung von SE am Universitätsklinikum Dresden zu erreichen, wurde ein spezielles Formular (Abb. 2) im Krankenhausinformationssystem ORBIS (damals Firma Agfa HealthCare GmbH) entwickelt und im Januar 2017 implementiert [15]. Bei der Entwicklung des Formulars wurden folgende Aspekte berücksichtigt: 1. Die Eingabe von Daten sollte möglichst einfach und anwenderfreundlich erfolgen, um eine hohe Nutzer-Compliance zu gewährleisten. 2. Die Daten werden in strukturierter Form erhoben, so dass sie auswertbar sind. 3. Die Kodierung basiert auf den international verwendeten ORPHAcodes der Orphanet-Database, welche mit weiteren Kodiersystemen wie ICD-10-WHO, OMIM, UMLS, MeSH und MedDRA verknüpft sind. Der in ORBIS hinterlegte Orphanet-Katalog wird halbjährlich aktualisiert, wobei eine hausinterne Meldung der Änderungen von Daten zwischen den Versionen implementiert wurde. 4. Das Formular ist verpflichtend an die Faktura-Freigabe gekoppelt, wodurch eine vollständige Erhebung stationärer Falle ermöglicht wird. Für den ambulanten Sektor war eine verpflichtende Kodierung nicht umsetzbar, da der Workflow in ORBIS hierfür keine technische Möglichkeit bot. 5. Um die klinikweite Sichtbarkeit insbesondere von Patient*innen mit multidisziplinärer Versorgung zu ermöglichen, ist das Formular in der Patientenhistorie hinterlegt. Auf diese Weise sind vorhandene Einträge für alle Abteilungen einsehbar, jedoch nicht editierbar. Neue Einträge werden fortlaufend vorhandenen Einträgen in einer Verlaufsdokumentation hinzugefügt, so dass Verdachtsdiagnosen, ausgeschlossene oder gestellte Diagnosen nachvollziehbar sind.

Nach Öffnen des Formulars wird als Erstes gefragt, ob eine Seltene Erkrankung vorliegt oder nicht (Abb. 2). Wird das Feld „Keine SE“ ausgewählt, ist keine weitere Aktion des Nutzers notwendig. Wird das Feld „Neu“ ausgewählt, öffnet sich eine Suchmaske mit den Feldern, „Orpha-Nummer“, „Bezeichnung/Synonym“, „ICD10“, „Gen“ und „Protein“, mit denen nach einer passenden Orpha-Kennnummer gesucht werden kann. Die Suchergebnisse werden alphabetisch sortiert angezeigt, wobei jeder angezeigte Datensatz sich für weitere Informationen expandieren lässt (Abb. 2). Die ausgewählte zu kodierende SE wird anschließend übernommen und muss durch die Angabe des Diagnosestatus „Gesichert“, „Verdacht“ oder „Ausschluss“ ergänzt werden. Erst dann kann das Formular validiert werden. Nachträgliche Änderungen sind nicht möglich, jedoch können durch das Anlegen eines neuen Formulars Änderungen in der Diagnose erfolgen. Alle Einträge zu Patient*in, einschließlich kodierender Klinik und Nutzer*innen, sind in der jeweiligen Patientenhistorie nachverfolgbar.

### Universitätsklinikum Würzburg

**Figure 3:**
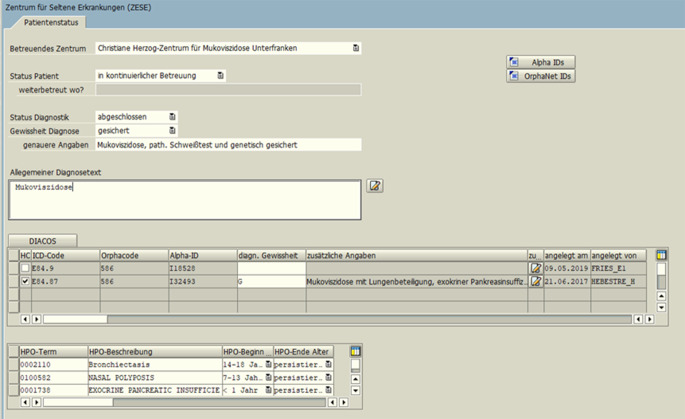

Die Kodierung nach ICD-10-GM erfolgt am Universitätsklinikum Würzburg mit Hilfe der Kodiersoftware ID DIACOS® im Krankenhausinformationssystem „SAP IS-H/i.s.h. med“. Um die Dokumentation wichtiger Informationen bei Patient*innen mit einer SE, wie u. a. ORPHAcodes, Alpha-IDs und HPO-Codes, zu ermöglichen, wurde im Jahr 2016 ein sogenanntes Parametrisiertes Dokument erstellt und seither weiterentwickelt (Abb. 3). Dieses Dokument kann händisch angelegt und befüllt werden, wird jedoch bei Kodierung einer Erkrankung, für die ein ORPHAcode mit dem ICD-10-GM-Kode verknüpft ist, automatisch angelegt und mit dem ICD-10-GM-Kode, der Alpha-ID und der Orpha-Kennnummer befüllt. Immer, wenn ein*e Patient*in erneut zur Behandlung am Universitätsklinikum Würzburg vorgestellt wird, wird die im Parametrisierten Dokument hinterlegte Kodierung der SE in das Diagnosemodul NDIA übernommen, unabhängig davon, ob eine ambulante, tagesklinische oder vollstationäre Betreuung stattfindet. Die Kodierassistent*innen und Ärzt*innen können dann entscheiden, ob die Erkrankung als Hauptdiagnose oder Nebendiagnose geführt wird oder ggf. sogar gelöscht werden muss.

Im Parametrisierten Dokument wird aktuell auch schon die Symptomatik des oder der Patient*in mittels HPO-Codes abgebildet. An der Erweiterung des Dokuments zur Dokumentation des gesamten „Set of Common Data Elements for Rare Diseases Registration“ der Europäischen Plattform für die Registrierung Seltener Erkrankungen wird aktuell gearbeitet.

### Universitätsklinikum Tübingen

**Figure 4:**
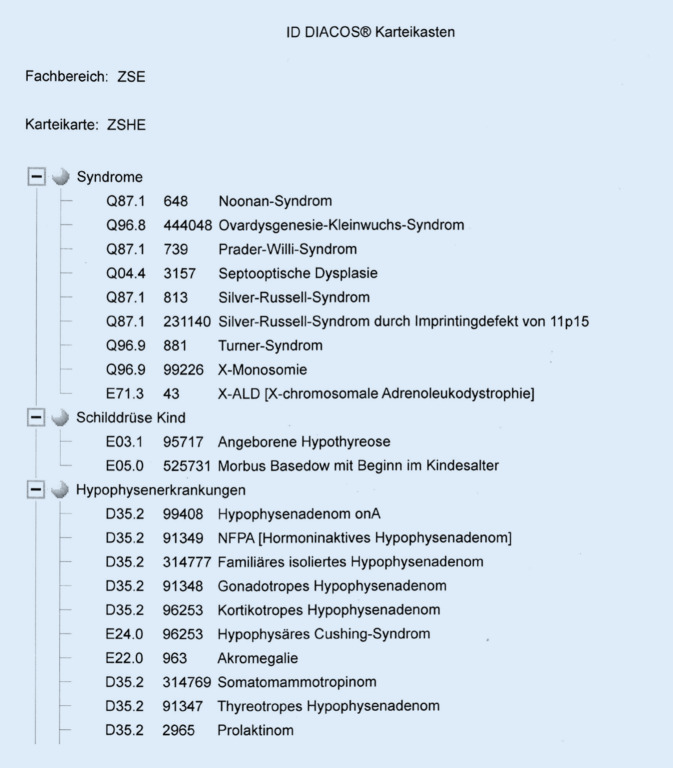

Am Universitätsklinikum Tübingen erfolgt in der IT-Umgebung des Krankenhausinformationssystems SAP IS-H/i.s.h. med die Kodierung nach ICD-10-GM mit Hilfe der kommerziellen Kodiersoftware ID DIACOS®. Klinikweit kann über ID DIACOS® auch ein entsprechender ORPHAcode für SE mitkodiert werden. Jedoch wurden im letzten Jahr 56 % der am Universitätsklinikum Tübingen in SAP kodierten ORPHAcodes über eine 2019 eigens für das Zentrum für Seltene Erkrankungen (ZSE) entwickelte hausinterne Lösung kodiert, da kommerzielle Anbieter erst seit Ende 2021 vergleichbare Möglichkeiten zur Orpha-Kodierung anbieten. Diese hausinterne Lösung stellt jedem Fachzentrum (Typ-B-Zentren des ZSE) eine spezifische, expertisebasierte Liste zur Verfügung, welche ausschließlich für das jeweilige Fachzentrum relevante ORPHAcodes kodiert. Mit dieser angepassten OE-Diagnosenhitliste, welche eine Zuordnung bevorzugter Diagnosen pro fachlicher Organisationseinheit bzw. Zentrum darstellt, können Diagnosen inklusive ORPHAcode direkt aus der Diagnosenmaske in SAP IS‑H heraus kodiert werden. Perspektivisch wird nun ein Übertrag dieser Listen in die sogenannten Karteikästen von ID DIACOS® dieselbe Funktionalität eines expertisebasierten Zugriffs auf eine einheitliche, vollständige und zentrumsspezifische Orpha-Kodierung am ZSE bereitstellen und die bisherige Lösung ersetzen (Abb. 4).

Seit 2020 hat die Anzahl der Orpha-kodierten Patient*innen Auswirkung auf die Finanzierung des jeweiligen ZSE-Fachzentrums, wodurch auch ein finanzieller Anreiz für die Orpha-Kodierung geschaffen wurde. Basierend auf dem Würzburger Parametrisierten Dokument (Abb. 3) soll zudem zeitnah eine weitere Maske zur Dokumentation am ZSE Tübingen implementiert werden, welche alle Elemente des „Set of common data elements for Rare Diseases Registration“ [14] der Europäischen Plattform für die Registrierung Seltener Erkrankungen abdeckt. Unter anderem kann dann die Symptomatik anhand von HPO erfasst werden. Eine Kodierung via SNOMED CT ist bisher nicht vorgesehen.

**Figure 5:**
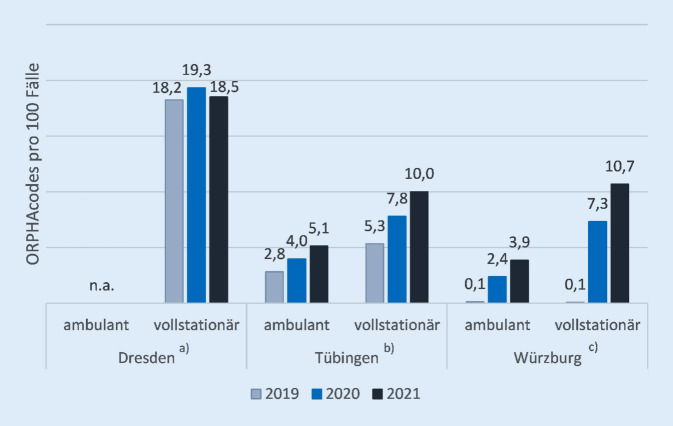

Die Entwicklungen der ORPHAcode-Kodierung ambulanter und stationärer Fälle an 3 Kliniken mit Zentren für SE ist in Abb. 5 dargestellt. Es ist hierbei im zeitlichen Verlauf von einem stabilen Verhältnis von Fällen mit SE zu solchen mit häufigen Erkrankungen auszugehen. Die dargestellte Entwicklung in Tübingen und Würzburg ist somit wahrscheinlich auf eine gesteigerte Dokumentation zurückzuführen.

## Diskussion

In den vergangenen zwei Jahrzehnten haben SE auch durch politische Maßnahmen deutlich an Sichtbarkeit gewonnen. Dies betrifft auch die Abbildung der SE in den verschiedenen Terminologien und Kodiersystemen. Eine zusätzliche Kodierung mit ORPHAcodes hat jedoch auch bei einer zukünftigen (hypothetischen) 100 %igen Abdeckung der SE bspw. über ICD-11 sowie durch das seit Oktober 2021 verfügbare SNOMED CT auf Orphanet-Mapping erhebliche Vorteile, auch im Hinblick auf die statistische Auswertung:

- SE sind in einigen Klassifikationssystemen nur Krankheiten unter allen Krankheiten, es gibt keinen konkreten Hinweis auf die Seltenheit einer Erkrankung. Alle SE werden zusammen mit häufigen Krankheiten klassifiziert, eine umfassende Analyse bestimmter Gruppen von SE ist somit ausgeschlossen, da relevante Zuordnungen fehlen. Diese Analysen sind jedoch mittels ORPHAcodes realisierbar und für weitergehende Maßnahmen, gerade im gesundheitspolitischen Kontext, unerlässlich. Sie können in Abhängigkeit von der Versorgungslage der Patient*innen wertvolle Informationen bspw. zum spezifischen Behandlungsbedarf liefern.
- Die Einbindung von Fachleuten mit einer besonderen Spezialisierung auf SE im Kurationsprozess der Orpha-Nomenklatur, insbesondere die Zusammenarbeit mit den European Reference Networks (ERNs), gewährleistet, dass die neuesten Erkenntnisse zeitnah „eingespielt“ werden und eine rasche Kodierung neuer Entitäten erfolgen kann. Ohne Nutzung der ORPHAcodes werden diese Erkenntnisse wohl nur zeitverzögert in die gängigen Kodiersysteme einfließen.
- Das umfangreiche Mapping mit anderen Ressourcen und Terminologien (auch unter Verwendung der HPO) erleichtert nicht zuletzt ganz wesentlich die grenzüberschreitende Interoperabilität mit anderen Systemen. Dies stellt eine wesentliche Voraussetzung dar, um den Datenaustausch nicht nur im Hinblick auf die gesamteuropäische Versorgung, sondern auch im Hinblick auf Grundlagenforschung zu ermöglichen.

Zur Implementierung der Orpha-Kodierung an den Standorten müssen einige Hürden genommen werden: Die (kommerziell) verfügbaren Kodier-Tools wurden z. T. noch nicht vollständig implementiert. Auch ist das Wissen um Notwendigkeit und Umsetzung von (korrekter) Orpha-Kodierung bei nichtärztlichen und ärztlichen Mitarbeiter*innen oft noch gering. Zudem kann sich der Kodiervorgang in der täglichen Praxis verlängern.

An mehreren Standorten in Deutschland wurde der Einsatz von Alpha-ID-SE und ORPHAcodes zur Abbildung von SE in den Krankenhausinformationssystemen bereits umgesetzt. Hierzu wurden verschiedene Institutions-individualisierte Lösungen entwickelt. Im Universitätsklinikum Dresden wird bei der Kodierung stationärer Fälle die Frage eingeblendet, ob eine SE vorliegt oder nicht. Zusammen mit einer anschließenden Unterstützung bei der Kodierung der Fälle mit SE durch ein strukturiertes Menü führt dieser Ansatz zu einer sehr hohen Rate von stationären Fällen mit einem erfassten ORPHAcode und damit zur Erkennbarkeit von SE in diesem Sektor. In Tübingen wird ein verhältnismäßig hoher Anteil an ambulanten Fällen mit ORPHAcodes erreicht, indem eine standardisierte und geführte Erfassung von SE in den Typ-B-Zentren die Eingaben erleichtert und die Kodierung von SE an die Finanzierung der Typ-B-Zentren gekoppelt ist. Die Einführung des patientenbezogenen Parametrisierten Dokuments zu SE in Würzburg erlaubt eine semiautomatische, ressourcenschonende Kodierung von SE mittels Alpha-ID und ORPHAcode. Dies gilt für alle Patient*innen, für die bereits in der Vergangenheit im Rahmen einer Behandlung der SE entsprechende Kodes erfasst wurden. Das Dokument hat zusätzlich den Vorteil, leicht um weitere relevante Datenfelder, wie zum Beispiel HPO-Codes, erweiterbar zu sein und auch nach Einführung z. B. der ICD-11 (ICD-11-GM) in der Zukunft die Kodierung von ORPHAcodes über vorhandene Alpha-IDs bei bekannten Patient*innen zu unterstützen.

Die drei dargestellten Ansätze sind grundsätzlich geeignet, eine vom Gesetzgeber geforderte Kodierung der Diagnosen stationärer Fälle mit SE anhand von entsprechenden Alpha-IDs und ORPHAcodes zu unterstützen. Eine Kombination der dargestellten Ansätze erscheint allerdings am ehesten geeignet, ressourcenschonend eine vollständige und korrekte Kodierung aller (stationären) Fälle mit einer SE zu erreichen. Hierzu sind zusätzlich entsprechende (wiederholte) Schulungen der kodierenden nichtärztlichen und ärztlichen Mitarbeiter*innen erforderlich.

## Ausblick

Die Erfassung von Diagnosen mit ICD-10-GM erfolgt in deutschen Krankenhäusern konform zu den Deutschen Kodierrichtlinien (DKR). Diese Richtlinien „sind ein Regelwerk, das primär die Abrechnung mit DRGs unterstützt“ [16]. Dies spiegelt sich in den Vorgaben zur Kodierung von Haupt- und Nebendiagnosen in den Kodierrichtlinien wider, die nur dann angegeben werden dürfen, wenn sie das Patientenmanagement beeinflussen. Es ist jedoch durchaus denkbar, dass ein Mensch mit einer SE z. B. aufgrund eines vielleicht unbekannterweise mit der SE in Verbindung stehenden Knochenbruchs stationär betreut wird, ohne dass das diagnostische oder therapeutische Management durch die SE unmittelbar beeinflusst wurde und ohne dass ein erhöhter Betreuungs‑, Pflege- oder Überwachungsaufwand bestand. In diesem Fall würde die SE auch in Zukunft nicht erfasst. Für epidemiologische Betrachtungen wäre auch in solchen Fällen die Schaffung einer Möglichkeit zur entsprechenden Kodierung anzustreben. Relevant wäre dies zudem beispielsweise für die Erfassung von bisher unbekannten Komorbiditäten und Komplikationen.

Die verpflichtende Kodierung von ORPHAcodes mit Hilfe der Datei Alpha-ID-SE bei stationären Fällen mit einer SE ab 2023 wird die Sichtbarkeit dieser Erkrankungen im Gesundheitswesen unzweifelhaft erhöhen. Menschen mit SE benötigen jedoch glücklicherweise nur relativ selten eine stationäre Aufnahme, die meisten Betroffenen können und sollen jahrelang rein ambulant betreut werden. Gerade mit Blick auf die hohen Kosten, die bei einer ambulanten Betreuung z. B. durch die komplexen multiprofessionellen Behandlungsbedarfe und/oder die medikamentöse Therapie entstehen, wäre für ein umfängliches Bild der SE im Gesundheitssystem eine möglichst spezifische und eindeutige Kodierung auch im ambulanten Bereich wünschenswert. Damit könnte dem speziellen Versorgungs- und Finanzierungsbedarf besser Rechnung getragen werden. Aktuell ist für den ambulanten Bereich die Kodierung der ORPHAcodes nur über die Anforderungen an Typ-B-Zentren des NAMSE adressiert [17] und wird damit für die Zertifizierung dieser Zentren relevant. Insgesamt kann also im ambulanten Bereich weiterhin nur eine freiwillige Kodierung der SE erfolgen. Die Schulungen und Informationen, die die Einführung im stationären Sektor begleiten, stehen aber allen Anwender*innen offen.

Für Menschen mit komplexen SE, ungewöhnlichen Phänotypen oder auch für noch undiagnostizierte Fälle ist die strukturierte und standardisierte Abbildung des Phänotyps zur Diagnosefindung und/oder Behandlung wichtig. Hier spielen gerade auch für die aktuellen Entwicklungen in Bezug auf innovative genetische Verfahren wie die aktuellen Selektivverträge zur Durchführung der Exom-Diagnostik und das Modellvorhaben zur umfassenden Diagnostik und Therapiefindung mittels Genomsequenzierung die Human Phenotype Ontology eine große Rolle. Die Möglichkeit zur Erfassung von HPO-Codes in den Krankenhausinformationssystemen und die Weitergabe an integrative Datenbanken wird damit zunehmend an Bedeutung gewinnen.

## Fazit

Die Kodierung stationärer Fälle mit SE anhand der Datei Alpha-ID-SE ist ab dem Jahr 2023 verpflichtend, sofern ICD-10-GM-Kode und ORPHAcode für die jeweilige SE enthalten sind. Dadurch wird die Sichtbarkeit von SE im deutschen Gesundheitssystem als Entscheidungsgrundlage für Krankenkassen, Leistungserbringer und die Gesundheitspolitik, aber auch als Basis für wissenschaftliche Arbeiten deutlich verbessert. Für ein umfängliches Bild der SE im Gesundheitssystem und um dem speziellen Versorgungs- und Finanzierungsbedarf besser Rechnung tragen zu können, wäre auch im ambulanten Bereich eine möglichst spezifische und eindeutige Kodierung wünschenswert. Für komplexe SE und bisher undiagnostizierte Patient*innen wird zusätzlich eine strukturierte Erfassung des Phänotyps benötigt.

## Footnotes

1 OMIM (Online Mendelian Inheritance in Man), https://www.omim.org/.

2 ICD-10-WHO (International Statistical Classification of Diseases and Related Health Problems, 10th Revision, World Health Organization), https://www.who.int/standards/classifications/classification-of-diseases.

3 UMLS (Unified Medical Language System), https://uts.nlm.nih.gov/uts/.

4 SNOMED CT (Systematized Nomenclature of Medicine Clinical Terms), https://www.snomed.org/.

5 MeSH (Medical Subject Headings), https://www.ncbi.nlm.nih.gov/mesh/.

6 MedDRA (Medical Dictionary for Regulatory Activities), https://www.meddra.org/.

7 Link zum Download u. a. der Alpha-ID-SE: https://www.bfarm.de/DE/Kodiersysteme/Services/Downloads/_node.html.

## References

[1] Marx MM, Dulas FM, Schumacher KM (2017). Verbesserung der Sichtbarkeit seltener Erkrankungen in Gesundheitssystemen durch spezifische Routinekodierung. Bundesgesundheitsblatt Gesundheitsforschung Gesundheitsschutz.

[2] Orphanet (2020) Procedural document: Orphanet nomenclature and classification of rare diseases. Version 02, March 2020. https://www.orpha.net/orphacom/cahiers/docs/GB/eproc_disease_inventory_R1_Nom_Dis_EP_04.pdf. Zugegriffen: 30. Apr. 2022

[3] Robinson PN, Mundlos S (2010). The human phenotype ontology. Clin Genet.

[4] Orphanet: an online rare disease and orphan drug data base. © INSERM 1999. http://www.orpha.net. Zugegriffen: 30. Apr. 2022

[5] Orphadata: Free access data from Orphanet. © INSERM 1999. http://www.orphadata.org. Zugegriffen: 30. Apr. 2022

[6] Rat der europäischen Union (2009f) Empfehlung des Rates vom 8. Juni 2009 für eine Maßnahme im Bereich seltener Krankheiten (2009/C 151/02). https://eur-lex.europa.eu/legal-content/DE/TXT/?uri=CELEX:32009H0703(02). Zugegriffen: 30. Apr. 2022

[7] Rath A, Olry A, Dhombres F, Brandt MM, Urbero B, Ayme S (2012). Representation of rare diseases in health information systems: the Orphanet approach to serve a wide range of end users. Hum Mutat.

[8] Aymé S, Bellet B, Rath A (2015). Rare diseases in ICD11: making rare diseases visible in health information systems through appropriate coding. Orphanet J Rare Dis.

[9] Action RD Steering, maintaining and promoting the adoption of Orphacodes across MS. joint action 677024 / RD-ACTION. http://www.rd-action.eu/workpackage/workpackage-5/. Zugegriffen: 30. Apr. 2022

[10] https://www.rd-code.eu/workpackage-4-implementation-in-member-states/. Zugegriffen: 23. Juni 2022

[11] Orphanet data for rare diseases. https://od4rd.eu/. Zugegriffen: 23. Juni 2022

[12] Nationales Aktionsbündnis für Menschen mit Seltenen Erkrankungen (2013) Nationaler Aktionsplan für Menschen mit Seltenen Erkrankungen. Handlungsfelder, Empfehlungen und Maßnahmenvorschläge. https://www.namse.de/fileadmin/user_upload/downloads/Nationaler_Aktionsplan.pdf. Zugegriffen: 30. Apr. 2022

[13] Das Bundesgesetzblatt im Internet (2021) Gesetz zur digitalen Modernisierung von Versorgung und Pflege (Digitale-Versorgung-und-Pflege-Modernisierungs-Gesetz – DVPMG). Bundesgesetzblatt Teil I Nr. 28 vom 8. Juni 2021, Das Bundesgesetzblatt im Internet.Bundesanzeiger-Verlag, Bonn. http://www.bgbl.de/xaver/bgbl/start.xav?startbk=Bundesanzeiger_BGBl&jumpTo=bgbl121s1309.pdf. Zugegriffen: 30. Apr. 2022

[14] European Platform on Rare Disease Registration (2022) Set of common data elements. https://eu-rd-platform.jrc.ec.europa.eu/node/31_de. Zugegriffen: 30. Apr. 2022

[15] Kretschmer T, Danker A, Müller O, Rösen-Wolff A, Lee-Kirsch MA, Berner R (2021). Wie häufig ist selten wirklich? Eine Erhebung zur Häufigkeit Seltener Erkrankungen an einem Universitätsklinikum. Gesundheitswesen.

[16] Deutsche Krankenhausgesellschaft (DKG), GKV-Spitzenverband, Verband der privaten Krankenversicherung (PKV), Institut für das Entgeltsystem im Krankenhaus (InEK GmbH) (2022) Deutsche Kodierrichtlinien. Version 2022. Seite VI. https://www.dkgev.de/fileadmin/default/DKR_2022.pdf. Zugegriffen: 27. Apr. 2022

[17] Nationales Aktionsbündnis für Menschen mit Seltenen Erkrankungen (NAMSE) Anforderungskatalog an Typ B Zentren (Fachzentren für Krankheit/Krankheitsgruppe x). Version 3.0, 12.04.2019. https://www.namse.de/fileadmin/user_upload/downloads/Anforderungskatalog_an_Typ_B_Zentren_120419.pdf. Zugegriffen: 21. Juni 2022

